# Molecular tracking and prevalence of the red colour morph restricted to a harvested leopard population in South Africa

**DOI:** 10.1111/eva.13423

**Published:** 2022-06-08

**Authors:** Laura Tensen, John Power, Gerrie Camacho, Raquel Godinho, Bettine Jansen van Vuuren, Klaus Fischer

**Affiliations:** ^1^ Institute for Integrated Natural Sciences, Zoology University of Koblenz‐Landau Koblenz Germany; ^2^ Department of Zoology, Centre for Ecological Genomics and Wildlife Conservation University of Johannesburg Johannesburg South Africa; ^3^ Directorate of Biodiversity Management, Department of Economic Development, Environment, Conservation and Tourism North West Provincial Government Mmabatho South Africa; ^4^ Mpumalanga Tourism and Parks Agency Nelspruit South Africa; ^5^ CIBIO, Centro de Investigação em Biodiversidade e Recursos Genéticos, InBIO Laboratório Associado Campus de Vairão, Universidade do Porto Vairão Portugal; ^6^ BIOPOLIS Program in Genomics, Biodiversity and Land Planning CIBIO, Campus de Vairão Vairão Portugal

**Keywords:** colour morphs, inbreeding, leopard, *Panthera pardus*, population genetics, *TYRP1* mutation

## Abstract

The red leopard (*Panthera pardus*) colour morph is a colour variant that occurs only in South Africa, where it is confined to the Central Bushveld bioregion. Red leopards have been spreading over the past 40 years, which raises the speculation that the prevalence of this phenotype is related to low dispersal of young individuals owing to high off‐take in the region. Intensive selective hunting tends to remove large resident male leopards from the breeding population, which gives young male leopards the chance to mate with resident female leopards that are more likely to be their relatives, eventually increasing the frequency of rare genetic variants. To investigate the genetic mechanisms underlying the red coat colour morph in leopards, and whether its prevalence in South Africa relates to an increase in genetic relatedness in the population, we sequenced exons of six coat colour‐associated genes and 20 microsatellite loci in twenty Wild‐type and four red leopards. The results were combined with demographic data available from our study sites. We found that red leopards own a haplotype in homozygosity identified by two SNPs and a 1 bp deletion that causes a frameshift in the tyrosinase‐related protein 1 (*TYRP1*), a gene known to be involved in the biosynthesis of melanin. Microsatellite analyses indicate clear signs of a population bottleneck and a relatedness of 0.11 among all pairwise relationships, eventually supporting our hypothesis that a rare colour morph in the wild has increased its local frequency due to low natal dispersal, while subject to high human‐induced mortality rate.

## 1 INTRODUCTION

Biodiversity loss is occurring at an alarming rate around the globe and across all trophic levels (Butchart et al., 2010). Arguably, the loss of apex predators has particularly strong effects on ecosystems (Estes et al., 2011). They are also one of the most challenging groups to conserve due to their large area requirements, low densities and reproduction rates, and conflicts with humans and livestock (Ripple et al., 2014). One large carnivore, the leopard (*Panthera pardus*), is increasingly persecuted across its range that has declined by 48–67% over the past few decades (Jacobson et al., 2016). The decrease in population numbers has been particularly prominent in South Africa (Swanepoel et al., 2016), where primary threats are habitat fragmentation, road mortality, snaring incidents, lack of natural prey, unsustainable hunting and poaching (Swanepoel et al., 2015). As a result, it is estimated that only 20% of South Africa is currently suitable for leopards (Swanepoel et al., 2013). Previous studies indicated that the above threats may also cause reduced reproductive output (Balme et al., 2009) and the loss of genetic diversity (Ropiquet et al., 2015; Uphyrkina et al., 2001), which further impacts population viability (Allendorf et al., 2008).

Large carnivores are particularly sensitive to the negative effects of hunting due to their inherently low genetic diversity (Ausband & Waits, 2020; Milner et al., 2007), resulting from lower population sizes (Frankham, 1996). Although leopards are believed to be genetically diverse compared with other large carnivores (Spong et al., 2000; Uphyrkina et al., 2001), local effective population sizes (i.e. the number of individuals reproducing and thus contributing to the gene pool) may have been drastically reduced as a result of high local off‐take in many populations (Balme et al., 2010; Naude et al., 2020; Pitman et al., 2015; Spong et al., 2000; Swanepoel et al., 2014). For instance, adult leopard mortality rates of over 50 per cent have been recorded outside protected areas in South Africa (Swanepoel et al., 2015). Intensive selective hunting also tends to remove large resident male leopards from the breeding population, which disrupts spatial population dynamics (Naude et al., 2020). High territory turn‐over and opportunistic male natal philopatry can further alter dispersal and metapopulation structure in leopards (Fattebert et al., 2015; Milner et al., 2007). This has also been illustrated in many other harvested large carnivores, such as pumas (*Puma concolor*; Newby et al., 2013), brown bears (*Ursus arctos*; Leclerc et al., 2017) and wolves (*Canis lupus*; Webb et al., 2011).

Population declines and the loss of genetic diversity through inbreeding and genetic drift might also have triggered the increasing occurrence of a rare coat colour dilution, referred to here as the red leopard. Coat colour is a prominent morphological feature in mammals and plays an essential role in their survival (Hubbard et al., 2010). Therefore, phenotypic variation and the direction of selection are crucial to understand the evolutionary potential of species and their persistence overtime (Assis et al., 2016). In wild cats, the adaptive function of coat patterns indicates that it mainly serves as camouflage (Caro, 2005; Eizirik et al., 2003; Ortolani & Caro, 1996). Felids show a wide variety in coat colouration and pattern, (Ortolani, 1999), which are genetically determined and shaped by environmental factors such as habitat, arboreality and nocturnality (Allen et al., 2011; Da Silva et al., 2016; Eizirik et al., 2010). Because coat colour is highly heritable, it is possible that genetic drift, in which alleles randomly become fixed in small populations, can affect phenotypic variability (Da Silva et al., 2016; Eizirik et al., 2010).

The prevalence of rare colour morphs, such as melanism, has been linked to disruptive selection, which occurs when a trait is favoured over the average phenotype in certain environments (Allen et al., 2011). Alternatively, colour morphs such as albinism or leucism can relate to a deficiency of heterozygotes (Fleck et al., 2016), which often occurs after a population bottleneck (Hedrick & Ritland, 2012). Thus, identifying the mechanisms that shape phenotypic variation in the wild can offer interesting insights into a species' response to environmental change and anthropogenic pressures (Gray & McKinnon, 2007). For instance, changes in the coat pattern of Eurasian lynx (*Lynx lynx*) became noticeable after a demographic bottleneck in the Carpathian Mountains, as a result of over‐harvest (Kubala et al., 2020). The loss of genetic diversity and increases of autosomal recessive alleles have also been linked to other colour polymorphisms in large cats, such as the king cheetah (*Acinonyx jubatus*; Kaelin et al., 2012), white lion (Cho et al., 2013) and golden tiger (*Panthera tigris*; Xu et al., 2013). Selective hunting for desirable traits can further influence frequencies of phenotypes in exploited populations, for instance resulting in reduced body size, slower growth rates and earlier sexual maturity (Allendorf & Hard, 2009).

**FIGURE 1:**
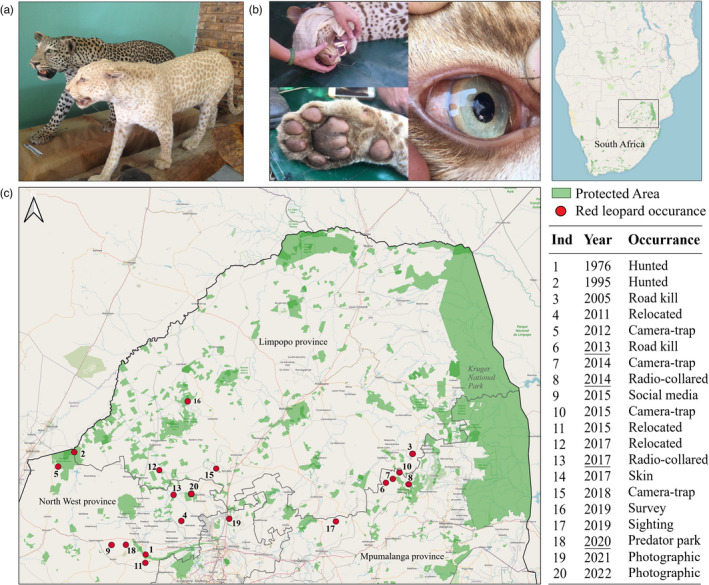
Colour pattern and distribution of red leopards. (a) The morphological difference between a normal and a red leopard (photograph by Laura Tensen). (b) Pink nose and paw pads related to the red leopard phenotype (photos by Derek van der Merwe). (c) Red leopard occurrence records in South Africa. Ind = individual. The year was underlined for individuals that were sequenced for this study

The red leopard phenotype, which was originally called a strawberry leopard (Dell'Amore, 2012), is a colour variant that, to the best of our knowledge, occurs only in South Africa. The colour morph is expressed by a general coat colour dilution, as animals exhibit a pale skin, faint spots, blue eyes, a pink nose and pink paw pads (Figure 1a,b). The phenotype is present at birth and persists throughout their life. Coat colour dilutions in big cats have been described as resulting from genetic variants in different genes, such as the agouti signalling protein (*ASIP*), melanocortin‐1 receptor (*MC1R*) or tyrosinase (*TYR*) protein‐coding gene (Cho et al., 2013; Eizirik et al., 2003; Schneider et al., 2012). The red leopard phenotype was originally described as being erythristic (Pirie et al., 2016), which has been well‐studied in domestic cats and results from the suppression of the black‐brownish pigmentation and the exclusive presence of pheomelanic pigments in the hair shafts, caused by mutations in tyrosinase‐related proteins (*TYRP)* (Schmidt‐Küntzel et al., 2005). We hypothesize that the recessive trait could also relate to leucism, in which white colour is caused by an absence of melanocytes in the skin (Cieslak et al., 2011). In golden tigers, however, which show the same phenotypic traits as red leopards, a mutation in the transporter protein *SLC45A2* and transmembrane serine protease *CORIN* drives the light pigmentation (Xu et al., 2013, 2017).

Twenty red leopards have been recorded between 1976 and March 2022, mainly in the North West and Mpumalanga provinces (Figure 1c). Red leopards have been increasingly sighted over the past 40 years, and during one survey in Mpumalanga's Thaba Tholo Wilderness Reserve, as much as 7% of the population (*N* = 28) had the red coat colour (Pirie et al., 2016). This observation adds to the speculation that the prevalence of this phenotype is related to genetic drift (Allendorf et al., 2008). Alternatively, the red coat colour dilution might result from disruptive selection, which can occur when colour morphs have selective benefits under specific environmental conditions, for instance by increased camouflage (Allen et al., 2011; Da Silva et al., 2017). Because phenotypic variation is an essential requirement for evolutionary change, it is of crucial importance to understand how it evolves in wild populations (Assis et al., 2016).

In this study, we aim to investigate the genetic mechanisms underlying the red coat colour morph in leopards. We also study red leopard demographics to understand the increased prevalence of this phenotype in South Africa and relate it to genetic parameters. The following hypotheses were tested: (i) the red leopard phenotype is the result of one or more non‐synonymous mutations in a coat colour associated gene (*MC1R*,*ASIP*,*TYR*,*TYRP1*,*SLC45A2* or *CORIN*), in accordance with the inheritance pattern in protein‐coding genes, (ii) the prevalence of the red coat colour morph is driven by increased relatedness in the population associated with high human‐induced mortality, and, accordingly, (iii) genetic diversity and the local effective population sizes are low. We have used an integrated approach that combines genetic data with population demographic and satellite‐telemetry data. This study is the first to investigate the genetic basis of the red leopard colour morph and will provide novel insights into population dynamics and evolutionary pathways. It will therefore contribute towards a better understanding of how colour morphs evolve in the wild and whether anthropogenic factors influence their occurrence and spread.

## 2 METHODS

### 2.1 Study area

This study was carried out in the northern part of South Africa, notably the North West Province (NWP) and the Mpumalanga Province (MP) (see Figure 1). The dominant vegetation biomes in both provinces is the savanna biome in the north and the grassland biome in the south (Mucina & Rutherford, 2006). They contain a number of vegetation types such as thornveld and mountain bushveld within the Central Bushveld bioregion. The NWP includes more arid regions in the west, that is the Kalahari, whereas in the east, the Drakensberg Escarpment extends into MP, with the Lowveld to the east (Mucina & Rutherford, 2006). The climate across the whole study area is mostly subtropical, with some temperate affinities on the southern margin of the bushveld region where the area is transitional with the Highveld plateau. Across all vegetation types, the mean annual rainfall ranges between 500 and 800 mm per annum, and falls mainly in summer. The monthly mean minimum and maximum temperatures range between −3.7 and 36°C, and the number of days with frost per year range between 3 and 28 days (Mucina & Rutherford, 2006).

Over thirty reserves are located in MP, among which are the Blyde River Canyon Nature Reserve (300 km^2^), Manyeleti Game Reserve (230 km^2^), Loskop Dam Nature Reserve (236 km^2^) and Songimvelo Game Reserve (480 km^2^). The southern part of Kruger National Park (Kruger, from here on) is also located in MP, which is the one of the oldest and largest protected areas in Africa (Carruthers, 1995), covering an area of over 19,000 km^2^. In the NWP, the main reserves are Madikwe Game Reserve (750 km^2^), Pilanesberg National Park (Pilanesberg, from here on) (550 km^2^), and the Magaliesberg Biosphere Reserve (3600 km^2^) which, although not a conventional nature reserve, still receives protection. The NWP and MP cover 76,490 km^2^ and 104,880 km^2^, respectively, of which only 34% and 12% are considered suitable habitat for leopards (Swanepoel et al., 2013). The NWP is estimated at 174 to 255 leopards, and the MP at 338–1851 individuals (Swanepoel, 2013; Swanepoel et al., 2014, 2016). Together, they hold 18% of the total leopard population of South Africa (Swanepoel et al., 2016).

### 2.2 Genetic sampling

Tissue samples were opportunistically obtained from leopards that were found dead in the field, and hair samples were collected when animals were immobilized for relocation or satellite‐collaring. No animal was exclusively immobilized for this study. A total of 39 samples was collected from various locations, mainly located in MP (see Table S1), and stored at −20°C. DNA was isolated using the NucleoSpin Tissue kit applying the support protocol for hair and tissue samples. DNA analyses were conducted at the University of Johannesburg. We first screened all samples by sequencing the mitochondrial (mtDNA) control region (see Table S2 for primer details). Low‐quality samples were removed from further analyses. In total, twenty normal leopards and four red leopards were used for further analysis.

### 2.3 Laboratory methods

The genomic exons (23 in total) of *MC1R*,*ASIP*,*TYR*,*TYRP1*,*SLC45A2* and *CORIN* were amplified in four red and four Wild‐type leopards and subsequently sequenced using a MultiGene Optimax Thermocycler (Applied Biosystems). To test the genotypic frequencies of any red leopard alleles, candidate genes of the remaining 16 leopards will be also be sequenced. The primer sets for candidate gene coding regions (see Table S3) were designed by Xu et al. (2013, 2017) based on the cat genome assembly (Felis_catus‐6.2). PCRs were performed in 20 μl reaction volumes containing 15 H_2_0 μl, 7.5 μl MyTaq polymerase (Meridian Bioscience), 0.5 μl MyTaq reaction buffer, 0.5 μl (25 nM) forward and reverse primer, and 1 μl DNA (20 ng). Thermal cycling conditions were performed with a MultiGene Optimax Thermocycler (Applied Biosystems) using the following PCR protocol: an initial 10 min at 94°C, 2 cycles of 30 s at 94°C, 30 s annealing starting at 60°C and decreasing 1°C every 2 cycles until reaching 50°C and 45 s at 72°C, 30 cycles of 30 s at 94°C, 30 s at 50°C and 45 s at 72°C, and a final extension at 72°C for 30 min. Sequencing products were separated by a commercial provider (Macrogen Europe B.V.). All novel sequences were added to the NCBI Genbank database (see Table S3).

We selected 20 nuclear microsatellite loci that are known to be polymorphic in South African leopards (Naude et al., 2020; Uphyrkina et al., 2001; see Table S3 for primer details), and genotyped all 24 leopards. Primers were 5′‐labelled with 6‐FAM, HEX, Tamra or TET fluorescent dyes. Polymerase Chain Reactions (PCRs) were performed in 10 μl volumes containing 3 μl (H_2_0), 5 μl (1×) Platinum Multiplex PCR Master Mix (Applied Biosystems), 0.5 μl (25 nM) of each primer, and 1 μl (~20 ng) genomic DNA. Amplifications were carried out in a Multigene Optimax Thermal Cycler (Labnet) with the following PCR cycling protocol: 3 min of initial activation at 93°C, 10 annealing cycles consisting of 94°C for 15 s, 52–56°C for 15 s and 72°C for 30 s, 20 annealing cycles consisting of 89°C for 15 s, 52–56°C for 15 s, 72°C for 30 s, and a final elongation step of 72°C for 30 min. PCR products were separated by electropohoresis in 2% agarose gel stained with GelRedTM (Biotium Inc., Hayward, CA, USA).

### 2.4 Statistical analysis

Sequence alignments were done in Geneious 6.1.5 (Kearse et al., 2012). Sequences of the candidate genes were aligned with mRNA sequences obtained from the NCBI Genbank database (Table S4), by multiple sequence alignment using ClustalW (Chenna et al., 2003). The candidate regions were scanned to search for the possible causal mutation that related to the red coat colour dilution. The coding regions were translated in amino acids using Geneious. SNPs between Wild‐type and red leopards that would introduce non‐synonymous substitutions or stop codons were identified as putative causal mutation(s) linked to the red morph. Synonymous SNPs were also considered, because they can disrupt splice‐donors downstream, potentially causing exon skipping (i.e. the removal from the mRNA) (O'Neill et al., 1998).

Microsatellite fragment scoring was done in Geneious (Kearse et al., 2012). For microsatellites, MICRO‐CHECKER v2.2 (Van Oosterhout et al., 2004) was used to test for genotyping errors and deviations from Hardy–Weinberg equilibrium, based on 1000 bootstraps and 95% confidence intervals (CIs). Linkage disequilibrium (LD) between all pairs of loci was assessed in Arlequin v3.5 (Excoffier & Lischer, 2010), with 10,000 permutations and a significance level fixed at 0.05. To assess genetic variability, Arlequin v3.5 (Excoffier & Lischer, 2010) was used to calculate the average number of alleles per locus (*A*), observed (*H*_O_) and expected (*H*_E_) heterozygosity, ± standard deviation (*SD*). Fstat v2.9 (Goudet, 2002) was used to calculate inbreeding coefficients (*F*_IS_). Pairwise relatedness between all individuals was calculated using ML‐relate (Kalinowski et al., 2006). The effective population size (*N*_e_), which is the ideal population size under which allele frequencies and heterozygosity values change at the same rate as the observed population, was estimated using NeEstimator v2 (Do et al., 2014). For this, we used the LD method for polygamous mating (Waples & Do, 2010) and parametric confidence intervals with critical value *p* = 0.05 and 95% CIs (Waples, 2006). LD is considered the most common and accurate method for small populations (Saura et al., 2015).

For inferring whether there is genetic structure present in our study population, we used structure v2.3 (Pritchard et al., 2000), which applies a Bayesian framework. Because of the low sample size and their limited demographic distribution, we applied both the admixture and no‐admixture for the ancestry of individuals, and assumed correlated allele frequencies (Hubisz et al., 2009). The program was run from *K* value 1 to 3 with 1,000,000 MCMC generations (discarding 10% as burn‐in) and 10 iterations. The mean estimate of the posterior probability of the data for a given *K* retrieved from Structure Harvester (Earl & vonHoldt, 2012) was used to determine the most likely number of genetic clusters. CLUMPAK was used to visualize the combined results of all runs into a histogram (Kopelman et al., 2015).

To explore whether the leopard population has gone through a recent bottleneck event, we used BOTTLENECK v1.2.02 (Piry et al., 1999), using the two‐phased (TPM) model. TPM tends to be the most appropriate mutation model for microsatellite loci (Di Rienzo et al., 1994). Because our microsatellite panel consists of dinucleotide perfect and imperfect repeats, we chose the proportions in favour of infinite alleles (IAM) over stepwise mutation (SMM). For the analysis, we applied 30% of SMM and 70% of IAM (Cornuet & Luikart, 1996). The computation was performed for the Wilcoxon test, which we ran for 1,000,000 iterations, and a variance set at 20. The Wilcoxon test provides relatively high power and can be used with any number of polymorphic loci (Luikart et al., 1998). The second test performed in BOTTLENECK was the allele frequency distribution test. This graphical method examines the frequencies of all alleles in a population and compares it to the distribution expected at mutation‐drift equilibrium. As rare alleles are rapidly lost after a bottleneck, the characteristic L‐shaped distribution of allele proportions at equilibrium no longer exists after a bottleneck (Luikart et al., 1998).

### 2.5 Demographic data

To give an indication of red leopard prevalence, we collected data on all reported occurrences from the literature (see e.g., Pirie et al., 2016), media reports and data kept by the provincial conservation authorities. We used a linear regression in Microsoft Excel to test whether occurrences of red leopards increased over time. We also obtained information of all leopards that were captured and handled (as part of human‐wildlife conflict mitigation, or repatriation of orphans) by the authorities of both the NWP and the Mpumalanga Tourism and Parks Agency (MTPA) since 2006 in the scope of management purposes. To measure human‐induced mortality, we used data and reports made available by NWP and MPTA. Mortality records from legal hunts were made available by relevant authorities. This only included events for which the cause of death was known, and thus excluded natural mortality. Nevertheless, by having recorded human‐induced mortality from 2006 to 2020, it allowed us to look at temporal trends. We divided the cause of death into four categories: (i) road kill; (ii) snared; (iii) hunted (legally or illegally); or (iv) other (e.g. poisoned, relocation‐related, unknown cause of death). All mortalities included in this study were confirmed by field biologists.

For spatial movement patterns, we used satellite‐telemetry data from three red leopards obtained from Power et al. (2021) in the NWP (*N* = 2) and G. Camacho (MPTA unpublished) in MP (*N* = 1). The leopards were fitted with dual VHF radio collars, with a Global Positioning System (GPS)/Iridum satellite (African Wildlife Tracking cc, 106 Nuffield Street, Rietondale, Pretoria). One collar was unique in that it was based on vehicle‐tracker technology and registered locations for every movement made (TractGroup, Unit 8, Block A, Blueberry Office Park, Apple Street, Honeydew, Johannesburg, 2040, South Africa). In NWP, two red male leopards were radio‐collared (between October 2017 and August 2018) as part of ongoing management purposes. In MP, one red male leopard was collared between January 2015 and June 2015. We recorded 170 daily locations during the study and observed the radio‐collared leopards on four occasions (for details, see Power et al., 2021). Decollaring was planned after a year of deployment by recapturing using cage traps placed at GPS clustered location sites. The movement data were plotted on a base map using Quantum GIS (QGIS) software, version 3.12, which was also used to calculate home‐range sizes with the minimum convex polygon (MCP, 95%) method.

## 3 RESULTS

### 3.1 Autosomal traits

**FIGURE 2:**
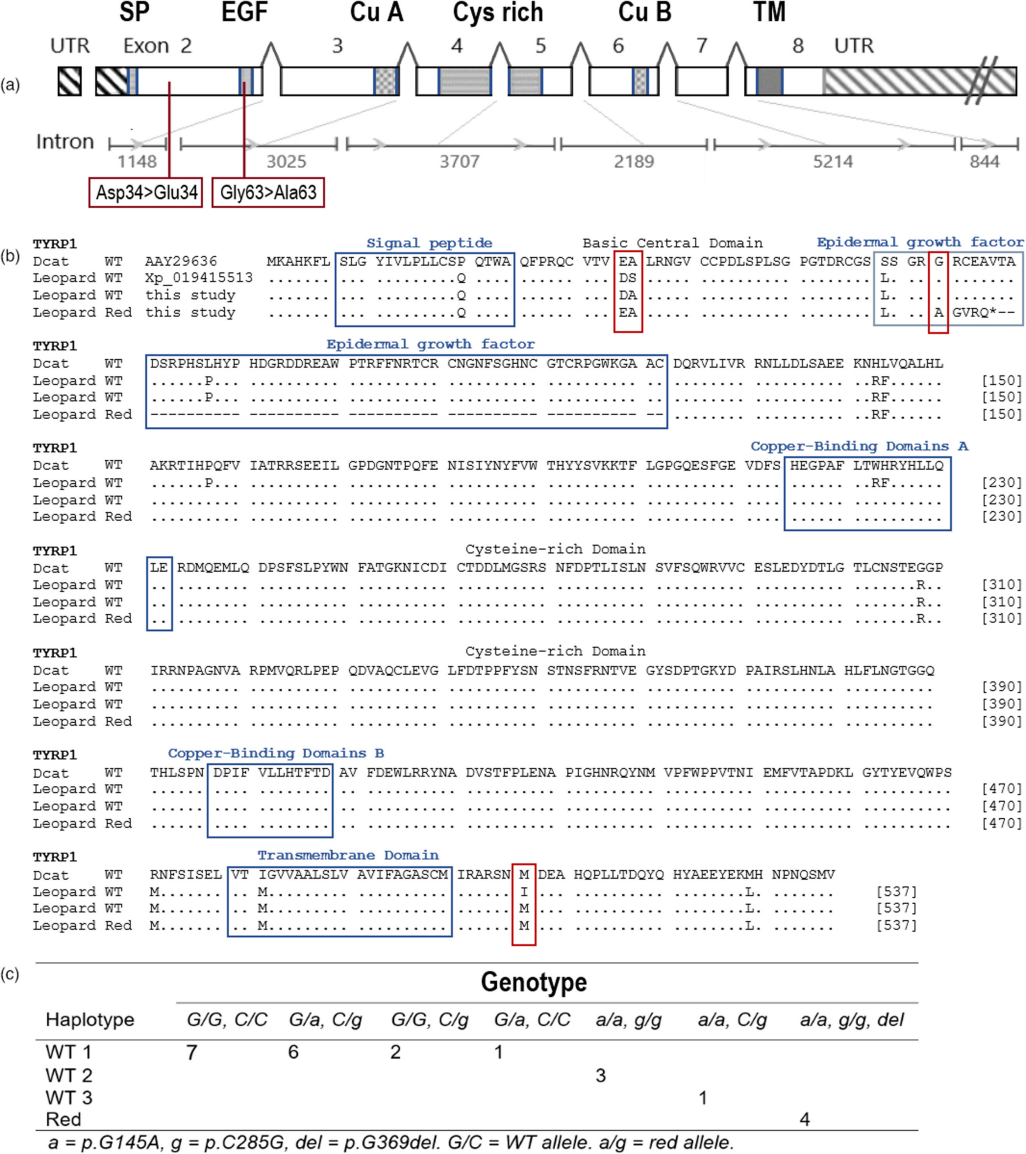
Identification of the red leopard associated mutations in the *TYRP1* gene. (a) Gene structure of *TYRP1* (adjusted from Schmidt‐Küntzel et al., 2005) with noncoding regions in dashed and the exons as boxes. The exons and domains are drawn to scale and annotated with genomic sequence NC_058380.1: SP = signal peptide, EGF = epidermal growth factor domain, (CuA, CuB) = copper‐binding domains A and B, Cys‐rich = cysteine‐rich domain, and TM = transmembrane domain. The red leopard specific amino acid mutations are highlighted in red, and the stop codon after Gly63 > Ala63 is indicated by *. (b) Polymorphisms of the *TYRP1* gene, distinguishing domestic cats (dcat), Wildtype (WT) leopard, and red leopard. Variable sites are highlighted, dots (.) represent residues identical to the mRNA protein sequence AAY29636, and dashes (−) indicate that this exon may be skipped. (c) Genotype frequencies of red leopard and WT alleles, resulting in four haplotypes

Of all 23 exons sequenced from six nuclear genes, exon 2 of the tyrosinase‐related protein 1 (*TYRP1*) gene was the only coding region that showed genetic variation specific to the 4 red leopards analysed, when compared with 4 Wildtype (WT) leopards. In total, we found one synonymous SNP, one non‐synonymous SNP and a single‐base pair deletion associated with red leopards in this exon (Figure 2a). The two SNPs occur in positions 145 (synonymous), and 286 (non‐synonymous) with reference to leopard *TYRP1* nucleotide sequence (NCBI XM_019415513). The SNP at position 286 resulted in an amino acid substitution at residues 34 with reference to leopard *TYRP1* protein sequence (NCBI XP_019271058), changing the aspartate to a glutamate (Asp34 > Glu34). These are both principial groups that act as Brønsted bases, conserving the overall protein functioning and charge, and are therefore not expected to induce major changes. Furthermore, the non‐synonymous mutation in *TYRP1* is not novel, but also found in domestic cats (NCBI AAY29636). The one base pair deletion occured in position 369, causing a frameshift in the *TYRP1* exon 2 after amino acid residue 63 (Gly63 > Ala63) and a stop codon at amino acid residue 68 (Figure 2b). The frameshift occurred in the domain that codes for the epidermal growth factor within the *TYRP1* gene. The two SNPs and the deletion were linked, representing one haplotype named red haplotype, while the other haplotypes were named WTs. Red leopards carried only the red haplotype (i.e. homozygous), while WT leopards carried both haplotypes. In the total study population, four WT leopards (17%) were homozygous for p.G145A, and three leopards (12.5%) were homozygous for p.C285G. Ten WT leopards were heterozygous for one or both alleles (Figure 2C).

We also found two non‐synonymous SNPs in *TYRP1* exon 2 and *TYRP1* exon 8 that were non‐specific to any phenotype. These consisted of a mutation at position 287, changing a serine to an alanine (Ser35 > Ala35), and at position 1705, which changed methionine to isoleucine (Met507 > Iso507), conserving the overall protein charge. Both SNPs are also found in domestic cats (Figure 2b). We also found genetic variation in *ASIP* exon 1, in which one non‐synonymous SNP at position 106 changed the amino acid at residue 36 from methionine to leucine (Met36 > Leu36) (see Figure S1). Both amino acids are neutrally charged. In the *TYR* gene, we found a non‐synonymous SNP in exon 5, changing a neutrally charged glutamine to a positively charged arginine in the protein sequence at residue 525 (Glu525 > Arg525, with reference to XP_019305593; Figure S1). We found no SNPs outside the coding regions (i.e. in the 5′ and 3′ UTR or introns).

### 3.2 Genetic diversity

The final data set consisted of 20 polymorphic microsatellite loci successfully scored for 24 leopards. Linkage disequilibrium between marker pairs was not observed. We found no evidence for stuttering and large allele dropout in our microsatellite data. When testing for Hardy–Weinberg equilibrium, homozygote excess was observed in 4 of 20 loci. We measured an average pairwise relatedness (*r*) of 0.11 ± 0.30 (*SD*), and found that 5.1% of the study population presented *r* > 0.2, which are half‐siblings. Notably, pairwise relatedness among the four red leopards was *r* = 0.16. The inbreeding coefficient was relatively high (*F*_IS_ = 0.19). The mean H_O_ and H_E_ values observed were 0.54 ± 0.08 and 0.66 ± 0.06, respectively. The average number of alleles per locus was 5.7 ± 1.2. The effective population size N_e_ was estimated at 56.8 (37.3–108.2) individuals. Significant bottleneck signals (*p* < 0.001) were found with the Wilcoxon test, and the allele distribution in the leopard population was shifted from normal L‐shaped allele frequencies to a mutation‐drift disequilibrium. We found no genetic structure in our data set, confirming that all leopards belong to the same population (*K* = 1; Figure S2).

### 3.3 Demographic data

**FIGURE 3:**
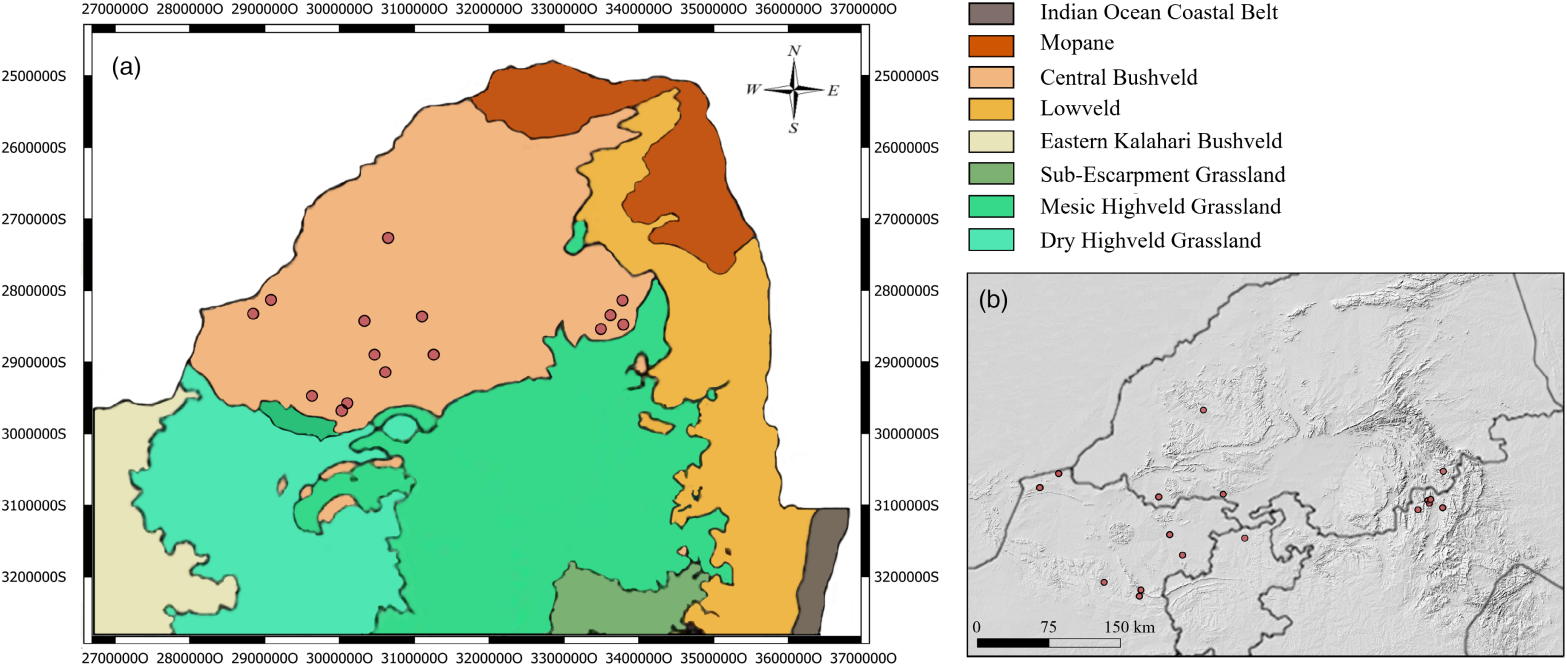
Red leopard occurrence. The red dots mark the red leopards that have been recorded between 1976 and 2021. (a) Bioregions of South Africa (based on Mucina & Rutherford, 2006). (b) A hill shade map of South Africa indicating terrain ruggedness (retrieved from the Stellenbosch University Digital Elevation Model)

Records of red leopards appear to be gradually increasing over the years (*R* ^*2*^ for the linear regression is 0.08, *p* = 0.32, based on Figure 1c), from one individual in 1976 to three in 2017 (Table S5). We found that red leopards made up ~3.6% of the study population (Table 1). In NWP, of 84 leopards that were captured and handled up to 2020, 2.8% were of the red phenotype. In MP, of 103 leopards that were handled, 4.4% were of the red phenotype. All reported occurrences of the red leopard morph were from the Central Bushveld bioregion (Figure 3a), and all records fall between latitudes 24° and 26° south. We have recorded red leopards in an additional two South African provinces, the Gauteng Province (GP) and Limpopo Province (LP) (Figure 3b). Furthermore, there is a large cluster of red leopards in NWP (i.e. 300 km diameter), which overlaps into GP and LP, while there is a tighter cluster in MP (<100 km diameter). Both clusters appear to be connected by ranges of hills and mountains (Figure 3b). We documented 149 human‐induced mortality records of leopards, i.e. on average 15 cases per year, which were mainly caused by hunting (62%) and snaring (18%) (see Table S6).

**TABLE 1:** Prevalence of the red coat colour morph among leopards that were captured and handled by authorities in the North West and Mpumalanga provinces in South Africa between 2011 and 2020

Year	North West			Mpumalanga		
	Wildtype	Red	%	Wildtype	Red	%
2011	11	1	8.3	5	0	0
2012	6	0	0	5	0	0
2013	5	0	0	3	1	25
2014	14	0	0	9	1	10
2015	8	1	11.1	10	1	9.1
2016	6	0	0	19	0	0
2017	10	1	9.1	26	0	0
2018	6	0	0	14	0	0
2019	13	0	0	7	0	0
2020	5	0	0	5	0	0
Average (±*SD*)			2.84 (±4.4)			4.41 (±7.8)

**FIGURE 4:**
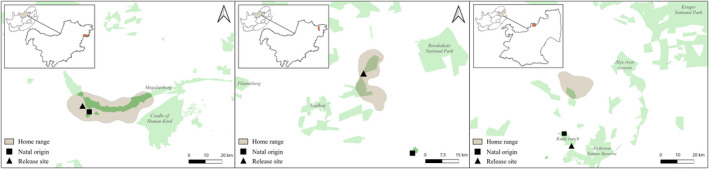
Home‐ranges of red leopards, showing the location of their natal origin and the release site after capture. GPS locations were retrieved from males in Magaliesberg (left), Beestekraal area (middle) in the North West Province, and the Lydenburg area (right) in the Mpumalanga Province

One collared red male leopard (NWP) was an orphan that was initially captured with a normal coat patterned brother. The second red male leopard was incidentally caught, and was subject to a short‐distance relocation within its home‐range (see Power et al., 2021 for further details). Interestingly, in 2022, another red leopard was discovered in its home‐range (Table S5). One of the individuals showed signs of natal philopatry, as he settled in the area where he was initially discovered as an orphan (Figure 4). The third (MP) red leopard dispersed from its natal origin and settled in a home‐range 30 kilometres further north. To avoid human‐wildlife conflict, it was recaptured and moved back to its natal territory. Following relocation, the leopard dispersed back to its northern home‐range, before suddenly disappearing. On the day that the radio‐signal was lost a helicopter was seen flying over the area, and suspicion was raised that the animal was caught and brought into a captive‐breeding facility. The average home‐range size for the three male red leopards was 350 km^2^.

## 4 DISCUSSION

The red leopard provides a unique opportunity to understand the molecular mechanisms of coat colour phenotypes in the wild, and gather information on the population dynamics of these colour morphs, especially in the face of anthropogenic pressures. We found one non‐synonymous mutation and a single‐base pair deletion associated with red leopards in the tyrosinase‐related protein (*TYRP1*) gene, which is referred to as the brown or ‘cinnamon’ locus (Kobayashi et al., 1998). This locus influences the development and maturation of melanocytes in the hair shafts, and the synthesis of melanin, thereby catalysing eumelanic pigments and mediating coat colour (Schmidt‐Küntzel et al., 2005). The deletion causes a frameshift in the amino acid sequence and introduces a stop codon, which likely affects the function of the protein, changing the melanogenesis pathway and triggering the appearance of the red phenotype. All four red leopards were homozygous for this mutation, while the Wild‐type individuals were either homozygous for the ancestral allele or heterozygous, which observation indicates that the red form is recessively inherited (Bauer et al., 2018). A previous study on mammalian melanogenesis has shown that *TYRP1* mutations can affect the catalytic functions of *TYR*, which is known as the albino loci (Schmidt‐Küntzel et al., 2005). Because *TYR* becomes down‐regulated in *TYRP1* mutant melanocytes, a reduced stability of *TYR* can cause the loss of pigments in cats (Kobayashi et al., 1998). Our results, therefore, indicate that red leopards are not erythristic, as was suggested by Pirie et al. (2016) and alluded toby Dell'Amore (2012), but likely linked to albinism pathways instead (Sarangarajan & Boissy, 2001).

The *TYRP1* protein is divided into four main regions: the signal peptide, an intra‐melanosomal domain, a transmembrane domain and a cytoplasmic domain (Li et al., 2017). The frameshift detected in our study (Met34 > Glu34) occurred in the intra‐melanosomal domain, and disrupts the epidermal growth factor, due which it is expected to have a large negative impact on *TYRP1* functioning. Furthermore, *TYRP1* is highly conserved among mammals, and this residue in particular has been conserved among vertebrate species ranging from fish to humans (Zhang et al., 2018). This supports the hypothesis that the Met34 > Glu34 mutation is disruptive and causes the red coat colour morph in leopards. Brown phenotypes are most common for *TYRP1* variants (Kobayashi et al., 1998), and exon 2 mutations in domestic cats have led to colour variants known as the chocolate and cinnamon cat (Lyon et al., 2005). Notably, the last sighting of a red leopard was of an individual much browner in colour (see Figure S3). It is possible that this individual is heterozygous for one of the red leopard alleles. We unfortunately did not have phenotypic data of heterozygous individuals, which took up 42 per cent of the population, to match our genotypic data, but this would be interesting to investigate in the future. In domestic cats, individuals that were heterozygous for the SNPs and a stop codon at amino acid 100, associated with the chocolate coat colour, resulted into the lilac Munchkin and chocolate silver Somali (Lyon et al., 2005).

Because the melanocortin 1 receptor (*MC1R*) and agouti signalling protein (*ASIP*) are most often related to pigmentation patterns in felids (Eizirik et al., 2003), our results illustrate that rare phenotypes in felids can occur through multiple pathways. We did find a mutation in the *ASIP* gene (Met36 > Leu36); however, this is not a novel SNP but commonly seen in mammals, including other Felidae species (Gershony et al., 2014). The black leopard phenotype is also caused by a SNP in the *ASIP* gene, which predictes amino acid change and induces the loss of gene function in exon 4 (Schneider et al., 2012), comparable to our red leopard mutation. Likewise, there was a significant association between melanism and a homozygous haplotype, consistent with a recessive mode of inheritance for this phenotype in leopards (Schneider et al., 2012). A white coat colour in Felidae species is typically caused by tyrosinase (*TYR*) mutational variants (Schmidt‐Küntzel et al., 2005). In lions, a single non‐synonymous SNP caused a white coat colour dilution (Cho et al., 2013). In our study, we also found a SNP in the *TYR* gene in leopards (Glu525 > Arg525); however, this is not novel and also occurred in Wild‐type lions. Animals with this variant do not appear to be phenotypically different (Cho et al., 2013).

Records of colour morphs are rare in the wild. For instance, the relative frequency of the ‘glacier’ bear (*Ursus americanus*) is 0.4% against 96.6% of black bears within southeast Alaska (Lewis et al., 2020). Four different coat colour morphs in sea lion (*Arctocephalus gazella*) on Livingston Island, Antarctica, occurred at a frequency of only 0.02–0.04% (Acevedo et al., 2009), and the ‘piebald’ white‐tailed deer in Florida, the United States, occurred at a frequency of <1% (Boughton et al., 2020). Rare wild cat variants such as the golden tiger, white lion and king cheetah have only been recorded a handful of times in history (Bottriell, 1987; Robinson & De Vos, 1982; Zebley et al., 1997). Red leopards, on the other hand, have been recorded more frequently and made up ~3.6% of the study population. The most frequent colour variant in leopards (i.e. melanism) although is more common, with an average frequency of 11% across its range (Da Silva et al., 2017). Habitat type is essential for the occurrence of melanistic leopards, where they are more common in tropical forests (30%) compared with open and dry biomes (0–6%). This indicates that melanism is strongly affected by natural selection, likely driven by the efficacy of camouflage and/or thermoregulation in different habitats (Majerus & Mundy, 2003).

Habitat‐dependent selection is common in mammals and influences phenotypic variation and cryptic colouration (Allen et al., 2011; Caro, 2005; Da Silva et al., 2016; Hoekstra et al., 2004). This raises the question of whether the high regional prevalence of red leopards is also the result of selection. It appears that this pigmentation is confined to the Central Bushveld bioregion, and that the records are within the same latitudinal zone (Mucina & Rutherford, 2006; Figure 3a). The Central Bushveld has historically showed strong cyclic patterns of alternating drier and cooler periods during the Late and Middle Pleistocene, between 19,000 and 23,000 years ago, when grassland readily replaced woodland (Scott, 1982; Scott et al., 2003). Afterwards, the red leopard phenotype, whether being interpreted as pale or red, could have arisen from background matching from a past state where open/arid grasslands prevailed in the vicinity of the occurrence records. Across carnivores, pale colour is associated with desert or semi‐desert environments, where concealment seems to have the most support (Caro, 2005; Ortolani & Caro, 1996). Notwithstanding, many felids in arid areas and open savannas are light tawny brown, such as lions (*Panthera leo*), or red, such as caracal (*Caracal caracal*) (Skinner & Chimimba, 2005), suggesting some background matching is already prevalent for nominate species. Even regional variation within species has been discovered, for example in the jaguarundi (*Puma yagouaroundi*), where it was convincingly shown that red forms of the species were associated with more arid and open ecosystems (Da Silva et al., 2016).

It is currently unknown whether the red coat has any sele ctive (dis)advantages, such as decreased (or increased) visibility by prey (Turner et al., 2015). Interestingly, the white‐phased Kermode or ‘spirit’ bear, which is a colour variant of black bears that occurs in a small portion of British Columbia in Canada, had a selective advantage during salmon capture (Klinka & Reimchen, 2009). Generally, coat colour variations such as albinism are considered a major disadvantage to animals in the wild, foremost due to decreased concealment and camouflage (Acevedo et al., 2009; Uieda, 2000). Albino animals are also disadvantaged due to a lack of pigment in the eyes, which is also observed in red leopards, because it causes visual impairment and makes it harder for animals to find prey (Miller, 2005). Furthermore, colour morphs are more frequent in small and isolated populations, or in populations that have recovered from a genetic bottleneck (Bensch et al., 2000; Holyoak, 1978). Based on our results, it is possibly that this has also been the case for red leopards, as we found signs of a small effective population size and a recent bottleneck event.

We therefore assume that the current prevalence of the red leopard phenotype in South Africa is caused by an increased relatedness of individuals in the area and eventually genetic drift (Kirkpatrick & Jarne, 2000), following a bottleneck event and the increase of the red rare variant frequency. Although dominant mutations are more likely to increase in populations than recessive mutations, significant genetic drift or inbreeding can increase the occurrence of rare colour morphs (Hedrick, 2011). Furthermore, recessive mutations are only purged out of a population if they have a strong selective disadvantage (Kirkpatrick & Jarne, 2000), which appears not to be the case for the red phenotype. Likewise, the high frequency of Kermode bears on Gribbell Island was attributed to restricted population size and isolation, and the relative effects of genetic drift (Hedrick & Ritland, 2012; Marshall & Ritland, 2002). The change in coat patterns of Eurasian lynx was also related to random fixation of alleles, after a recent bottleneck that resulted from over‐harvest (Kubala et al., 2020).

Although leopards were considered to be numerous in South Africa, and a high effective population size is reported continentally (Pečnerová et al., 2021), the regional population sizes in many parts of the country is likely to have been reduced due to anthropogenic pressures (Balme et al., 2010; Swanepoel, 2013; Swanepoel et al., 2015). There has recently been a strong increase in leopard snaring (6–13% of the population, Swanepoel et al., 2015). Another threat is retaliatory killing by livestock and game owners, which seems to have increased since the introduction of the hunting quota regulation and leopard hunting moratorium in 2016, where landowners instigate illegal killings out of spite (Mann et al., 2018). In total, we have recorded 149 cases of unnatural mortality between 2006 and 2020. Even though we have only recorded unnatural mortality, this tends to outweigh natural mortality. In Phinda Private Game Reserve, South Africa, Balme et al. (2009) found that more leopards were killed by humans (*N* = 19) than those that died of natural and unknown causes (*N* = 12). On a national scale, Swanepoel et al. (2015) found that within unprotected areas 95% of leopard mortality was accounted for by humans. The leopard mortalities recorded during our study were also primarily outside protected areas.

The NWP, where records of red leopards have first occurred (1976; Table S5), is characterized by a low density of leopards (Mann et al., 2018). An annual harvest rate of 0.4 leopards/100 km^2^ was determined (Thorn et al., 2012), and this density equals the leopard density in some reserves in NWP, such as Madikwe (Mann et al., 2018). In the MP cluster, an off‐take of 3–5 leopards/100 km^2^ has been reported (Pirie, 2016), which is not sustainable. Over‐harvest is known to disrupt the spatial organization in carnivores due a high population turn‐over and increased home‐range vacancies (Frank et al., 2018; Newby et al., 2013). This reduces the necessity for young males to disperse long distances in search of a home‐range, which affects both local and large‐scale metapopulation dynamics (Blyton et al., 2015). This could also explain why red leopards have not yet successfully moved into the nearby Kruger and Pilanesberg, where no record is known to date (see Bailey, 1993; Van der Merwe et al., 2020). Interestingly, the first record of the red leopard, in 1976, was one farm away (<10 km) from where we report recently on a male residing in a stable home‐range (Power et al., 2021). There is thus a 40‐year period, or 3–4 generations (after Swanepoel et al., 2016), over which the red leopard has occurred in the locality. This pattern is common for recessive genetic traits that are of recent origin (Hedrick & Ritland, 2012).

In our study, we found evidence that one male red leopard settled in the area where it was born, clearly illustrating a case of natal philopatry (Figure 4). The other two leopards dispersed <30 km away from their natal ranges, which is a tenth of what they are capable of (Fattebert et al., 2013). So even though our study population might not be physically isolated, natal philopatry is likely to reduce landscape linkage and ultimately promote inbreeding in leopards (Naude et al., 2020). This was supported by the presence of close familiar relationships (*r* > 0.15) in our random population sample. The level of observed and expected heterozygosity (H_O_/H_E_) were also found to be lower in our study population (0.54/0.66) compared with Kruger (0.70/0.74; Ropiquet et al., 2015), and instead were more comparable to a population in Phinda‐uMkhuze (0.65/0.70) that also suffers from unsustainable anthropogenic mortality (Naude et al., 2020).

In conclusion, this study illustrates a rare colour dilution in the wild associated with a recessive haplotype in the *TYRP1* gene. The increasing appearance of this colour morph may be related to the high population turn‐over which has also increased natal philopatry. For future research, it would be interesting to better understand the genetic underpinning of the red leopard polymorphism. First, whole‐genome data could allow us to estimate the presence and strength of selection in this genomic region. It would also allow us to estimate the age of the red allele, which could support our hypothesis that the red leopard prevalence is of recent origin. We also would like to highlight that red leopards might become targeted by trophy hunters (Milner et al., 2007), or otherwise predator breeders. After one of our study animals disappeared, suspicion had been raised that it was illegally caught and brought to a captive‐breeding facility. The red leopard is likely to be more financially valuable than normal coated animals (Nowers, 2018), so they may be intrinsically more threatened. By protecting red leopards, it would hopefully be possible to conserve this rare and unique predator in the wild.

## CONFLICT OF INTEREST

The authors declare that there is no conflict of interest.

## BENEFIT‐SHARING STATEGMENT

A research collaboration was developed with scientists from the countries providing genetic samples, and all collaborators are included as co‐authors. The results of this research will be shared with the provider communities and the broader scientific community. The research addresses a priority concern, in this case the conservation of leopards.

## Supporting information


Appendix S1

## Data Availability

The sequence data have been made available via the online repository Genbank (ON159784‐87). The microsatellite data have been made available via the online repository Dryad (https://doi.org/10.5061/dryad.j0zpc86h9).
