# Supplementary material for: Molecular tracking and prevalence of the red colour morph restricted to a harvested leopard population in South Africa
Source: Evol Appl. 2022 Jun 8;15(6):1028–41. doi: 10.1111/eva.13423 (PMC9234631; doi:10.1111/eva.13423)

**Table S1**. Leopard samples from South Africa utilized for this study. On the right side of the table are genotype frequencies of the four haplotypes we found in *TYRP1* exon 2, based on two SNPs (p.G145A and p. C286G) and one deletion (p.369del).

| **#** | **Code** | **Date** | **Sample** | **Sex** | **Location** | **Province** | **Latitude** | **Longitude** | **Haplotype** | **SNP1** | **SNP2** | **Deletion** |
| --- | --- | --- | --- | --- | --- | --- | --- | --- | --- | --- | --- | --- |
| 1 | Lp1 | 2017 | Tissue | F | Middelburg | MP | - | - | WT 1 | G/G | C/C |  |
| 2 | Lp2 | 2015 | Hair | M | Loskop Mooikopje | MP | -25.51198 | 29.28223 | WT 2 | a/a | g/g |  |
| 3 | Lp5 | 2015 | Blood | M | Rietvaley | MP | -24.92298 | 30.37103 | Red | a/a | g/g | Deletion |
| 4 | Lp6 | 2011 | Tissue | F | Watson Thaba Thol | MP | -24.96145 | 30.35328 | WT 1 | G/G | C/C |  |
| 5 | Lp9 | 2012 | Tissue | M | Adu Kudu Ranch | MP | -24.99193 | 30.38962 | WT 1 | G/a | C/g |  |
| 6 | Lp10 | 2016 | Tissue | M | Adu Kudu Ranch | MP | -24.98826 | 30.38929 | WT 1 | G/a | C/g |  |
| 7 | Lp11 | 2014 | Tissue | M | Adu Kudu Ranch | MP | - | - | WT 1 | G/a | C/g |  |
| 8 | Lp14 | 2013 | Tissue | F | Sekhukune rd | MP | -25.03469 | 30.22622 | Red | a/a | g/g | Deletion |
| 9 | Lp17 | 2016 | Tissue | F | Loskopdam | MP | - | - | WT 1 | G/G | C/C |  |
| 10 | Lp18 | 2017 | Hair | M | Breytenbachkraal | MP | -24.95513 | 30.37103 | WT 1 | G/G | C/C |  |
| 11 | Lp20 | 2013 | Tissue | F | Lydenburg | MP | - | - | WT 2 | a/a | g/g |  |
| 12 | Lp22 | 2017 | Blood | M | Breytenbachskraal | MP | -24.95496 | 30.66121 | WT 1 | G/G | C/g |  |
| 13 | Lp24 | 2017 | Blood | M | Klipfontein | MP | -24.98729 | 30.46819 | WT 1 | G/a | C/C |  |
| 14 | Lp25 | 2016 | Blood | F | Breytenbachskraal | MP | -24.57225 | 30.39399 | WT 1 | G/a | C/g |  |
| 15 | Lp28 | 2017 | Tissue | F | Rietvalley | MP | - | - | WT 2 | a/a | g/g |  |
| 16 | Lp36 | 2018 | Tissue | - | Ceyton Pass | MP | - | - | WT 1 | G/G | C/C |  |
| 17 | Lp37 | 2018 | Tissue | M | Next to Sabi Sand | MP | -24.71651 | 30.41083 | WT 1 | G/a | C/g |  |
| 18 | Lp39 | 2018 | Blood | F | Phalaborwa mine | MP | -23.95978 | 31.10508 | WT 3 | a/a | C/g |  |
| 19 | Lp40 | 2017 | Hair | M | Jabulani ranch | NWP | -25.17061 | 27.63549 | Red | a/a | g/g | Deletion |
| 20 | Lp41 | 2018 | Blood | - | Groenkraalfontein | MP | -25.5654 | 25.5654 | WT 1 | G/G | C/C |  |
| 21 | Lp46 | 2019 | Tissue | F | Malelane | MP | -25.45451 | 31.55370 | WT 1 | G/G | C/C |  |
| 22 | Lp47 | 2019 | Tissue | M | Skukuza | MP | -24.94917 | 31.50133 | WT 1 | G/G | C/g |  |
| 23 | Madiba | 2019 | Hair | M | Predator Park | NWP | - | - | Red | a/a | g/g | Deletion |
| 24 | Liyana | 2019 | Hair | F | Predator Park | NWP | - | - | WT 1 | G/a | C/g |  |

**Table S2.** Mitochondrial and nuclear primers used during this study.

| **Locus** | **Forward primer (5’–3’)** | **Reverse primer (5’–3’)** | **Product size (bp)** | **Reference** |
| --- | --- | --- | --- | --- |
| L0, E3 | CCCAAAGCTGAAATTCTACTTAAACTA | ATGACCCTGAAGAAAGAACCAG | 298 | Xu et al., 2013 |
| MC1R-ex-a | CATTGTGCCTGAGCTGACAT | GTCTCCAGCACACTGCTCAC | 429 | Xu et al., 2013 |
| MC1R-ex-b | CTGCACTCGCCCATGTATTA | CACCAGCATGGCTACAAAGA | 399 | Xu et al., 2013 |
| MC1R-ex-c | TCGCCTACTACGATCACACG | GCCATAGGATATCCCCACCT | 472 | Xu et al., 2013 |
| ASIP-ex1 | TCATCAGTTCCCCTTTCCTG | GGGAGCACGTTTGACATCTT | 418 | Xu et al., 2013 |
| ASIP-ex2 | CTCTTCTCCCACACCCTGAG | CCCTTAGCTCTCTGGGCTTC | 348 | Xu et al., 2013 |
| ASIP-ex3 | AGAGTTCCCAAGGCTGAGGT | AAATGCAAGAAAGCCACACC | 450 | Xu et al., 2013 |
| TYR-ex1-a | GCAGGAGAGATGCAGAGGAG | TTGGAGGGTCTGAAATCTGG | 366 | Xu et al., 2013 |
| TYR-ex1-b | TGTTCCCACAGCAACAAGAA | TTTGGCAACTTCATGGGATT | 386 | Xu et al., 2013 |
| TYR-ex1-c | TCGCTTCTCTGTGCAGTTTG | GATCCGTGAAGACGAGGGTA | 371 | Xu et al., 2013 |
| TYR-ex2 | CATGATTCAGAAAAACAGAAGAAA | TCTCTAGCCACAACTCCTTTAACA | 310 | Xu et al., 2013 |
| TYR-ex3 | TCTCTAGCCACAACTCCTTTAACA | TTTCAACTACCAGGGCCAAC | 356 | Xu et al., 2013 |
| TYR-ex4 | TTGGCATCCTTCCATGTCTT | GGCTTTGGGGATAGCATTGT | 298 | Xu et al., 2013 |
| TYR-ex5 | TAGCAGAGCTGGCATTCAAA | AAAATGAGGTCAACCTTGTTGG | 423 | Xu et al., 2013 |
| TYRP1-ex1 | GGGAAGGGAATCATGTGCTA | CATTCGTTAGCAGGTCATGG | 399 | Xu et al., 2013 |
| TYRP1-ex2 | CATTCGTTAGCAGGTCATGG | TGTACATGCAGATCCCCACT | 511 | Xu et al., 2013 |
| TYRP1-ex3 | GTGGCACATTTTCACGTTTG | CTGTCGGGGACCTGAAAAG | 399 | Xu et al., 2013 |
| TYRP1-ex4 | CAGGGAGAAAACCAAGCAAA | TGAGGACTTTCAGACAGCACA | 355 | Xu et al., 2013 |
| TYRP1-ex5 | TTCTGTAAGTGTTCCTATACCCTGT | TGTGACAACTAGATAAAATCAAAGCA | 249 | Xu et al., 2013 |
| TYRP1-ex6 | AGACATTGGCAACGGTTTTC | CCATACCACCTGAACGGTCT | 289 | Xu et al., 2013 |
| TYRP1-ex7 | TTAACAAGATGTCTTTGGCATATTT | TTTTGGTAATTTCCACAGAAAAGA | 217 | Xu et al., 2013 |
| TYRP1-ex8 | TGTTCTCCTTTTGTTTTTCACAG | CAGAGAATTGGTCGTTTGTCA | 250 | Xu et al., 2013 |
| SLC45A2-ex7 | TGT TCC TGT GTT TCC CTT GC | CCCTTCACTGTGTTGGAGGT | 288 | Xu et al., 2013 |
| CORIN exon13 | TGC TTC CAG GTT TTC TGT CC | TCC TAG CCA GGC ATT AAC GTA | 214 | Xu et al., 2017 |

* Xu, X., Dong, G.X., Hu, X.S., Miao, L., Zhang, X.L., Zhang, D.L., Yang, H.D., Zhang, T.Y., Zou, Z.T., Zhang, T.T., & Zhuang, Y. (2013). The genetic basis of white tigers. *Current biology*, *23*(11), 1031-1035.

**Table S3.** Microsatellite markers used during this study.

| **Multiplex pool** | **Locus** | **Forward primer (5’–3’)** | **Reverse primer (5’–3’)** | **Product size (bp)** | **Florescent label** | **Annealing temp. (°C)** |  |
| --- | --- | --- | --- | --- | --- | --- | --- |
| 1 | FCA008 | ACTGTAAATTTCTGAGCTGGCC | TGACAGACTGTTCTGGGTATGG | 122 – 148 | HEX | 56 |  |
| 1 | FCA026 | GGAGCCCTTAGAGTCATGCA | TGTACACGCACCAAAAACAA | 136 – 154 | Tamra | 56 |  |
| 1 | FCA043 | GAGCCACCCTAGCACATATACC | AGACGGGATTGCATGAAAAG | 116 – 128 | 6-FAM | 56 |  |
| 2 | FCA075 | ATGCTAATCAGTGGCATTTGG | GAACAAAAATTCCAGACGTGC | 103 – 143 | 6-FAM | 54 |  |
| 3 | FCA077 | GGCACCTATAACTACCAGTGTGA | ATCTCTGGGGAAATAAATTTTGG | 143 – 155 | 6-FAM | 56 |  |
| 2 | FCA090 | ATCAAAAGTCTTGAAGAGCATGG | TGTTAGCTCATGTTCATGTGTCC | 93 – 120 | HEX | 56 |  |
| 4 | FCA094 | TCAAGCCCCATTTTACCTTC | CACCTGAGCCAAAGGCTATC | 215 – 237 | 6-FAM | 56 |  |
| 2 | FCA097 | TAATGTTCAACTTGAATTGCTTCC | GAACAGTAGTTTGCCCATACAGG | 138 – 148 | Tamra | 56 |  |
| 1 | FCA105 | TTGACCCTCATACCTTCTTTGG | TGGGAGAATAAATTTGCAAAGC | 189 – 197 | TET | 52 |  |
| 4 | FCA126 | GCCCCTGATACCCTGAATG | CTATCCTTGCTGGCTGAAGG | 139 – 145 | 6-FAM | 56 |  |
| 4 | FCA139 | AGCATGTTTTTGAGGCAGCT | TTATGGGTAGTGTGAAGTAGGG | 141 – 147 | Tamra | 56 |  |
| 5 | FCA161 | TTACCGATACACACCTGCCA | CACAGACGTGCTCTAGCCAA | 179 – 187 | 6-FAM | 56 |  |
| 2 | FCA211 | TGTAGAACATAATGCCTCAGCC | TCTTGAACCTATTTCCCCACA | 111 – 119 | TET | 52 |  |
| 3 | FCA220 | CGATGGAAATTGTATCCATGG | GAATGAAGGCAGTCACAAACTG | 214 – 222 | HEX | 56 |  |
| 3 | FCA224 | CTGGGTGCTGACAGCATAGA | TGCCAGAGTTGTATGAAAGGG | 154 – 162 | Tamra | 52 |  |
| 3 | FCA229 | CAAACTGACAAGCTTAGAGGGC | GCAGAAGTCCAATCTCAAAAGTC | 160 – 170 | TET | 52 |  |
| 4 | FCA247 | GGAAATTAGGAGCTCTGCCA | AAGATTTACCCAGTTGCCCC | 145 – 151 | TET | 52 |  |
| 5 | FCA310 | TTAATTGTATCCCAAGTGGTCA | TAATGCTGCAATGTAGGGCA | 121 – 137 | TET | 52 |  |
| 5 | FCA453 | AATTCTGAGAACAAGCTGAGGG | ATCCTCTATGGCAGGACTTTG | 186 – 198 | Tamra | 56 |  |
| 5 | FCA678 | AGCAATCTCCAGAATGTGTGG | TCAAAAGATTAAAGCCTTCCAA | 226 – 234 | HEX | 56 |  |
|  | | |  |  |  |  |  |

**Table S4**. Accession numbers of nucleotide/protein sequences downloaded from and admitted to Genbank

| **Gene** | **Leopard** | **Downloaded from** | **Submitted to** |
| --- | --- | --- | --- |
| ASIP | Wildtype | JX845175 / AGB56419 | - |
| MC1R | Wildtype | AY237396 / AA062313 | - |
| TYR | Wildtype | XM_019450048 / XP_019305593 | - |
| TYRP1 | Wildtype 1 | XM_019415513 / XP_019271058 | ON159784 |
|  | Wildtype 2 | - | ON159785 |
|  | Wildtype 3 | - | ON159786 |
|  | Red | - | ON159787 |
| SLC45A2 | Wild | XM_019450689 / XP_019306234 | - |
| CORIN | Wild | XM_019412711 / XP_019268256 | - |

**Table S5.** The total number of red leopard individuals recorded in South Africa.

| **Ind** | **Year** | **Sex** | **Age** | **Observer** | **Location** | **Province** | **Latitude** | **Longitude** | **Notes** |
| --- | --- | --- | --- | --- | --- | --- | --- | --- | --- |
| 1 | 1976 | Unknown | Unknown | Allan Retief | Retief farm | North West | -25.80942° | 27.27040° | Hunted, photograph available |
| 2 | 1995 | Unknown | Unknown | Anon* | Botswana border | Limpopo | -24.64991° | 26.41167° | Hunted |
| 3 | 2005 | unknown | Unknown | B. van der Waal* | R36 provincial road | Mpumalanga | -24.73323° | 30.56872° | Roadkill |
| 4 | 2011 | Female | 3-4 years | John Power | Sable Ranch | North West | -25.45902° | 27.74077° | Relocated to Borakalalo; returned to near Sable ranch, where she died in a farmer’s cage trap |
| 5 | 2012 | Male | 2-3 years | Dell' Amore* | Madikwe Game Reserve | North West | -24.81670° | 26.21670° | Sightings and camera-trap images |
| 6 | 2013 | Female | Adult | Anon* | Sekhukhune road | Mpumalanga | -25.03469° | 30.22622° | Road kill |
| 7 | 2014 | Female | Adult | Pirie et al. 2016 | Thaba Tholo Wilderness | Mpumalanga | -24.98322° | 30.35086° | Camera-trap images |
| 8 | 2014 | Male | 2-3 years | G. Camacho | Lydenburg area | Mpumalanga | -25.21428 | 30.49640 | Satellite collaring |
| 9 | 2015 | Unknown | Unknown | Anonymous | Koster Dam area | North West | -25.71948° | 26.89476° | Social media. It appeared that the individual was moved to a captive facility (#18?) |
| 10 | 2015 | Unknown | < 1 year | Pirie et al. 2016 | Thaba Tholo | Mpumalanga | -24.93310° | 30.33716° | Camera-trap images |
| 11 | 2015 | Male | < 6 months | Vasti Botha | Olifantshoek | North West | -25.85667° | 27.27551° | Captured on camera trap, orphaned. Caught and released after 2 years from captivity, and released in natal range. |
| 12 | 2017 | Female | 1-2 years | Derek van der Merwe | Thabazimbi | Limpopo | -24.91027° | 27.49065° | Captured and relocated |
| 13 | 2017 | Male | 3-4 years | Vasti Botha | Jabulani ranch | North West | -25.17061° | 27.63549° | Captured and radio-collared |
| 14 | 2017 | Unknown | Adult | Annelize Badenhorst Kotze | Bobonong | Botswana | -24.10258 | 27.82899 | Skin without tag. Alleged to have been obtained in the area or in South Africa |
| 15 | 2018 | Male | 3-6 years | Kelly Marnewick | Zwartkloof | Limpopo | -24.87148° | 28.16190° | Camera-trap images |
| 16 | 2019 | Male | < 7 years | Gareth Mann | Kaingo Game Reserve | Limpopo | -24.10258° | 27.82899° | Obtained during Panthera's surveys via camera traps |
| 17 | 2019 | Female | Adult | James Speedy | Klipnek, Loskop valley | Mpumalanga | -25.44840° | 29.61172° | Sighting, though no photographic record is availed. |
| 18 | 2020 | Male | 2-3 years | Nazir Cajee | Akwaaba Predator Park | North West | -25.71282° | 27.08781° | Captive animal, origin unknown, perhaps progeny #9? |
| 19 | 2021 | Male | 2-3 years | Dawid Boshoff | Dinokeng | Gauteng | -25.37859° | 28.30915° | Photo taken from helicopter. The same individual seems to have been camera-trapped subsequently in 2022. It is darker in colour than other red leopards (see Fig S3). |
| 20 | 2022 | Female | 2-3 years | Francois Van Breda | Ngala Kulala, Buffelspoort | North West | -25.15929 | 27.63036 | Photo- and videographic evidence was obtained, and it is possible that it could be a young male. Owing to proximity, may be progeny of #13. |

* *In*: Pirie, T.J., Thomas, R.L., & Fellowes, M.D. (2016). Erythristic leopards Panthera pardus in South Africa: short notes. *Bothalia-African Biodiversity & Conservation*, *46*(1), 1-5.

**Table S6**. Human-induced mortality among leopards in the North West and Mpumalanga province. Mortality cases that were listed as ‘other’ were also human-induced, such as poisoning or relocation.

|  | **North West** | | | |  | **Mpumalanga** | | | |
| --- | --- | --- | --- | --- | --- | --- | --- | --- | --- |
| *Year* | *Road kill* | *Snared* | *Hunted* | *Other* |  | *Road kill* | *Snared* | *Hunted* | *Other* |
| 2006 |  |  | 2 |  |  |  | 2 |  |  |
| 2007 |  |  | 5 | 1 |  | 1 |  |  |  |
| 2008 |  |  | 7 | 1 |  | 2 |  |  |  |
| 2009 |  |  | 10 |  |  | 1 | 1 |  |  |
| 2010 |  |  | 8 |  |  | 1 |  | 3 |  |
| 2011 |  |  | 7 | 1 |  |  | 3 | 2 |  |
| 2012 |  | 1 | 10 |  |  | 1 | 1 | 2 |  |
| 2013 |  | 1 | 10 |  |  | 3 |  | 2 | 2 |
| 2014 |  | 2 | 7 | 4 |  |  | 4 | 3 | 2 |
| 2015 |  | 1 | 5 | 1 |  | 3 | 1 | 2 |  |
| 2016 | 1 |  |  |  |  | 1 | 4 | 3 |  |
| 2017 |  | 1 |  | 1 |  |  | 1 |  |  |
| 2018 |  | 1 |  |  |  |  |  | 3 | 1 |
| 2019 |  | 2 |  |  |  |  |  | 1 | 2 |
| 2020 |  | 1 |  |  |  | 2 | 1 | 3 |  |
| Total | 1 | 10 | 71 | 9 |  | 14 | 17 | 22 | 5 |

**Figure S1.** Polymorphisms in leopard (*Panthera pardus*) of the ASIP gene, distinguishing two Wildtypes and two colour morphs (black leopards and red leopards) (Schneider et al, 2012), and of the TYR gene, distinguishing three Wildtype leopards, Wildtype lions (*Panthera leo*) and white lions (Cho et al, 2013).

**
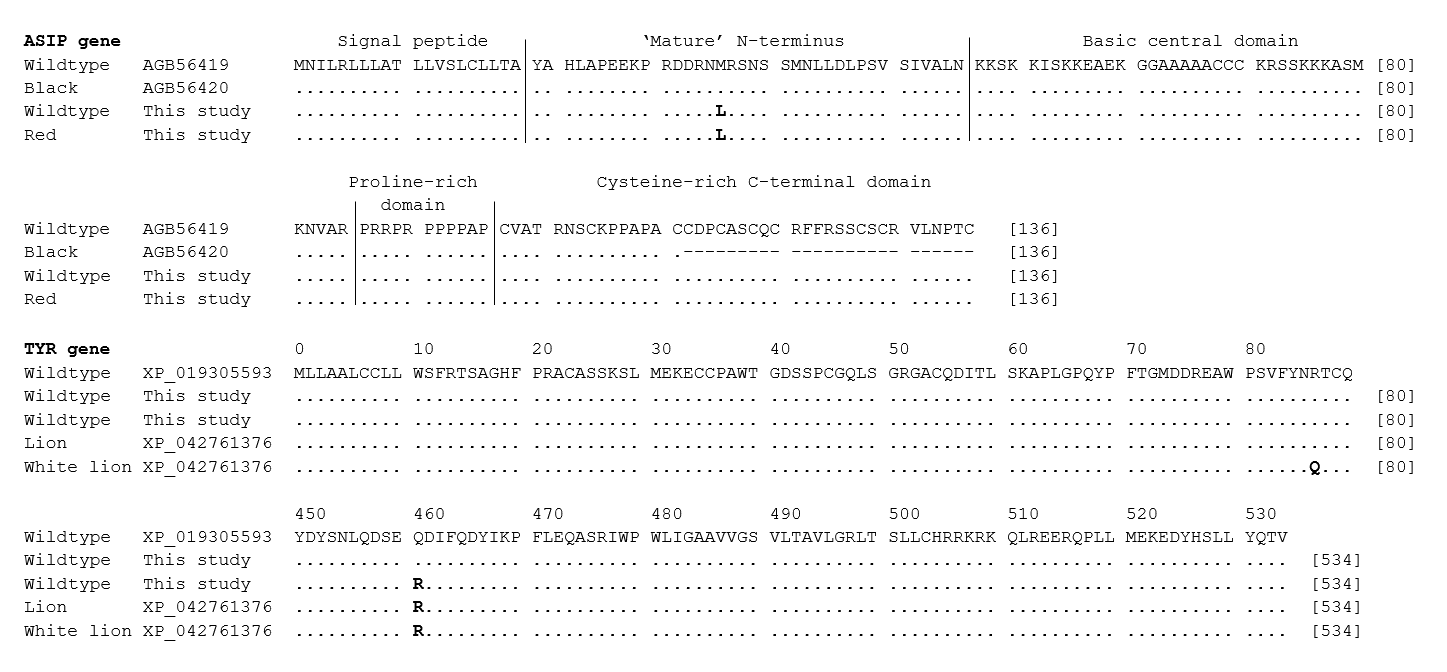
**

**Figure S2.** CLUMPAK results based on STRUCTURE analysis, illustrating that the leopards sampled in this study are part of one population.


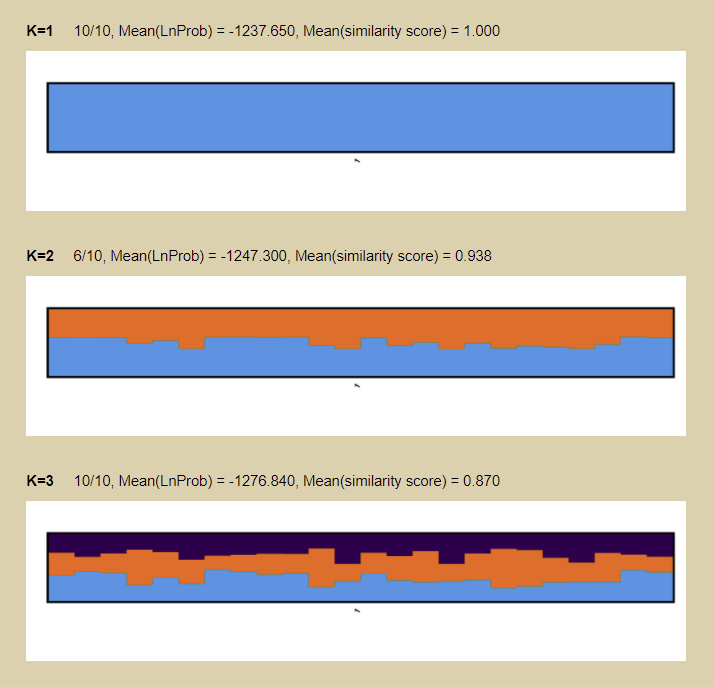


**Figure S3.** A sighting of a red leopard with a darker brown coat colour (left; photo by Dinokeng Management) compared to other red leopards (middle; photo by Francois van Breda) and Wildtype leopards (right; camera trapped by Graham Murray).


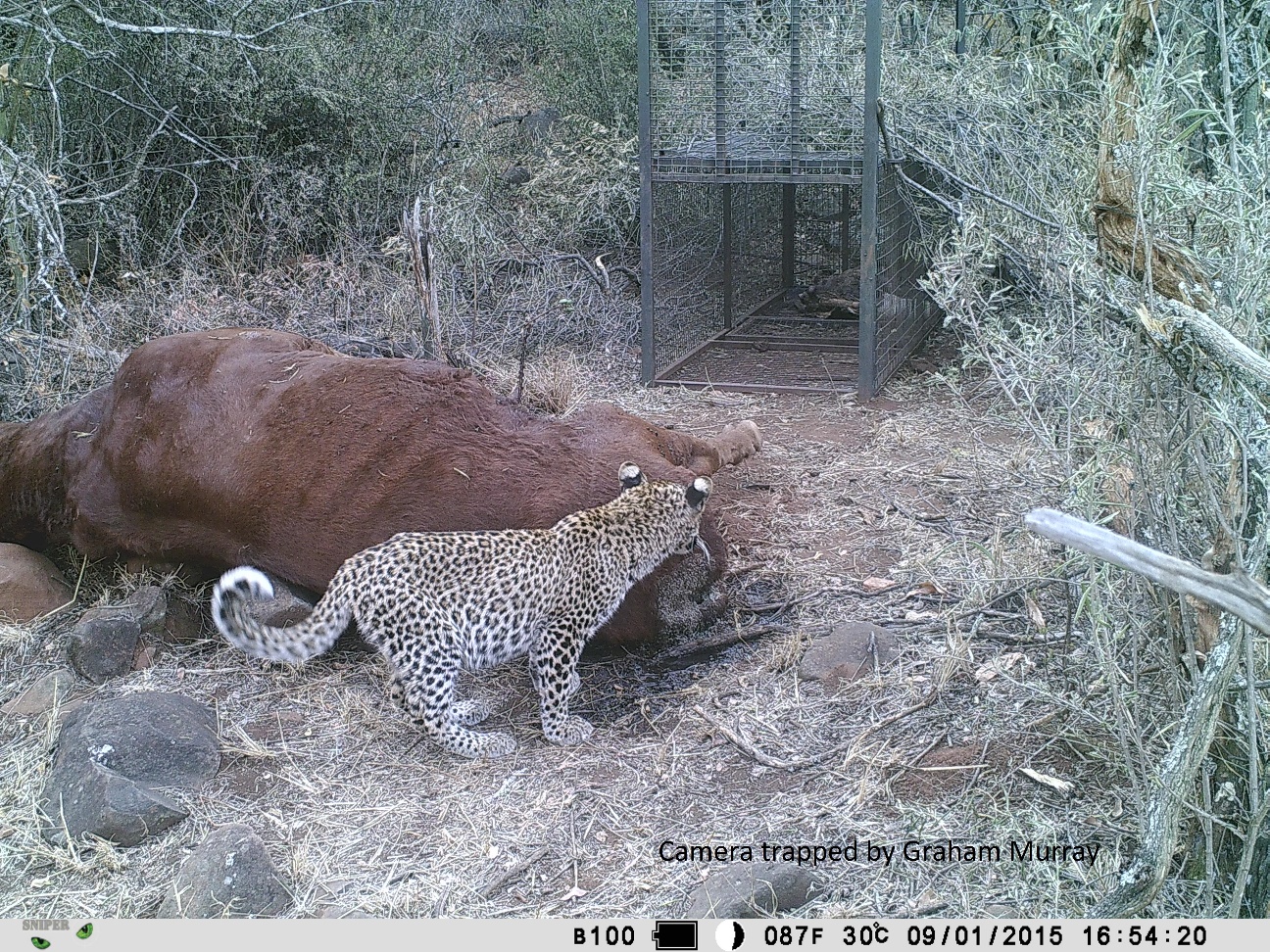

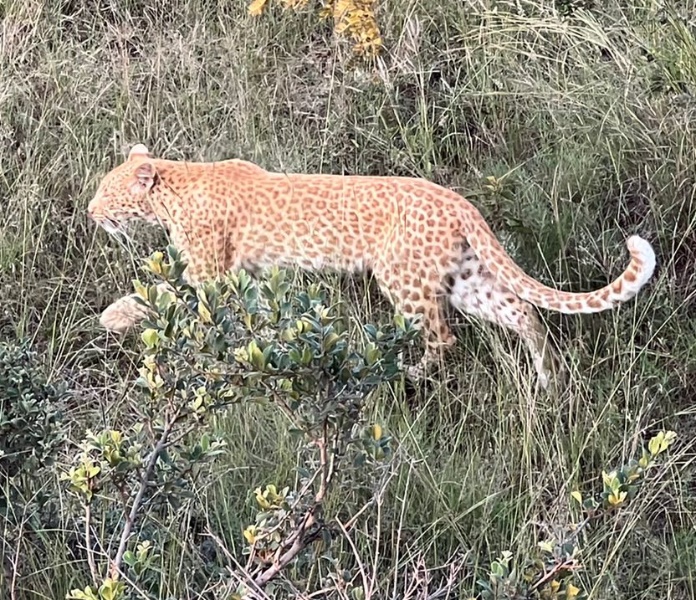

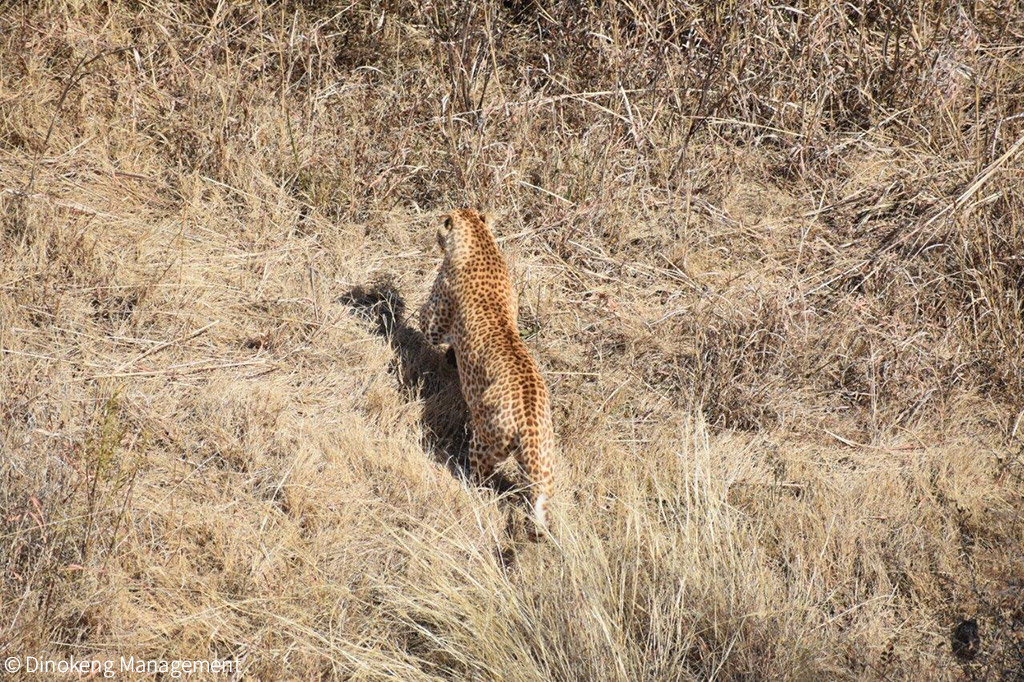

Supplement: Supplementary file 1 — Appendix S1 [file EVA-15-1028-s001.docx]
